# Engineered LDH-alginate composite hydrogel columns for selective heavy metal removal

**DOI:** 10.3389/fchem.2025.1649831

**Published:** 2025-08-07

**Authors:** Hualai Hao

**Affiliations:** Department of Plant Engineering, Sichuan Vocational and Technical College of Architecture, Deyang, China

**Keywords:** composite hydrogel, adsorbent, heavy metal ions, heavy metal removal, solid phase extraction

## Abstract

**Introduction:**

Heavy metal pollution poses significant food safety risks. To address this, a composite hydrogel composed of hydrotalcite and alginic acid was developed for adsorbing cationic heavy metal pollutants prevalent in food-related wastewater.

**Methods:**

The composite hydrogel was synthesized via hydrothermal methods and chemically crosslinked with calcium ions. Its adsorption capacity for representative cationic pollutants (Cu^2+^, Zn^2+^, Pb^2+^, Cd^2+^, Mn^2+^) was evaluated using Atomic Absorption Spectrometry (AAS). Cr^3+^ and Cr(VI) were excluded due to their anionic speciation, incompatible with the hydrogel’s cation-targeted adsorption mechanisms. Adsorption experiments were complemented by isotherm fitting and kinetic analyses.

**Results:**

The composite hydrogel exhibited the highest affinity for Cu^2+^ with a maximum adsorption capacity of 325.73 mg/g, followed by Zn^2+^ and Pb^2+^ at 284.78 mg/g. The adsorption process conformed to the Langmuir isotherm model and pseudo-second-order kinetics.

**Discussion:**

The composite hydrogel demonstrated significantly superior adsorption performance compared to individual hydrotalcite and alginate hydrogels. This enhanced capability indicates its promising potential for application in heavy metal remediation, particularly for mitigating food safety risks associated with cationic metal contaminants.

## 1 Introduction

The widespread contamination of water resources by toxic heavy metals (e.g., Pb, Cu, Cd, Zn, Mn) remains a pressing global concern, primarily driven by industrial and agricultural activities. These persistent, non-biodegradable pollutants pose serious risks to aquatic ecosystems and human health due to their bioaccumulative nature and capacity to induce cellular damage, neurological disorders, and organ failure—even at trace concentrations (Jiang et al., 2019; Jiang et al., 2020; Lin et al., 2021; Pavithra et al., 2021; Wu et al., 2021; Zhang et al., 2023; Zhao et al., 2019). As such, the development of efficient removal technologies, particularly adsorbents with selective capture and regeneration capabilities, is urgently required.

Hydrogel-based adsorbents have emerged as promising candidates owing to their tunable porosity, high surface functionality, and reusability. Among these, mineral–polymer composites offer unique advantages by combining the structural stability and ion-exchange properties of inorganic matrices with the processability and functional versatility of organic polymers. Hydrotalcite (LDH) clays, in particular, are attractive due to their layered structure, anion-exchange capacity, and intrinsic affinity for specific cations (Arshad et al., 2019; Rodrigues et al., 2019). In contrast, alginic acid biopolymers are rich in carboxyl groups that enable strong complexation with metal ions (Li et al., 2021; Lu et al., 2020). Strategically integrating LDH and alginate yields a composite system with potential synergistic benefits. However, the underlying mechanisms governing heavy metal uptake in such systems—especially the interplay between interlayer anion exchange and carboxyl coordination—remain insufficiently explored (Feng et al., 2022; Guo et al., 2023; Li et al., 2024; Zhu et al., 2023).

Despite growing interest in composite hydrogels, limited research has examined their performance in food-related water systems, where multiple cationic pollutants coexist and compete for adsorption sites. Moreover, real-world contamination scenarios often involve moderately toxic but commonly encountered ions such as Cu^2+^, Zn^2+^, and Pb^2+^, rather than rarer or highly regulated species like Cr^6+^. Consequently, targeted investigations focusing on the selective removal of these representative ions are both scientifically significant and urgently needed from a food safety perspective.

Although numerous studies have addressed hydrogel composites for water remediation, several key limitations persist. First is the issue of material synergy: most existing work focuses either on single-component systems (e.g., pure alginate) or composites containing unrelated fillers such as graphene oxide (Mehrabi et al., 2021) or bentonite (Tordi et al., 2025). Comparative studies that systematically evaluate standalone hydrotalcite, standalone alginate, and their composites—critical for confirming synergistic effects—are scarce. Second, the complexity of multi-ion systems remains largely unaddressed. Existing evaluations predominantly focus on single-metal systems or synthetic effluents, overlooking the competitive adsorption behavior that typifies real wastewater containing coexisting ions such as Cu^2+^, Zn^2+^, and Pb^2+^ (Mao and Gao, 2021; Wei et al., 2022). Third, practical performance metrics—such as adsorption capacity across varied concentrations, stability under pH fluctuations, and regeneration over multiple cycles—require further investigation to assess the suitability of these materials for real-world deployment (Campea et al., 2021; Gong et al., 2021; Omidian et al., 2024).

To address these gaps, this study investigates a food-safe LDH–alginate composite hydrogel under conditions that mimic multi-ion wastewater scenarios. The composite exhibits improved performance across several critical parameters and demonstrates strong potential for application in food safety monitoring and environmental protection.

In terms of adsorption capacity, the LDH–alginate composite hydrogel achieves a maximum uptake of 325.73 mg/g for Cu^2+^, significantly outperforming both standalone hydrotalcite and alginate hydrogels. This value is notably higher than those reported for other composite hydrogels, which range from 87.2 mg/g for Cu^2+^ to 2026.87 mg/g for Pb^2+^, depending on material composition and testing conditions.

Regarding selectivity, the composite hydrogel displays a clear preference for Cu^2+^ and Zn^2+^ over Pb^2+^, Cd^2+^, and Mn^2+^ in competitive multi-ion environments. The observed selectivity trend—Cu^2+^ > Zn^2+^ > Pb^2+^ > Cd^2+^ > Mn^2+^—is more pronounced than that of other hydrogels, such as chitosan/calcium alginate/bentonite composites, which exhibit weaker differentiation among ion types. This high selectivity is essential for effective remediation in complex wastewater matrices.

In terms of reusability, the composite hydrogel maintains high adsorption efficiency over multiple adsorption–desorption cycles, with minimal performance degradation. This marks a substantial improvement over many other composite hydrogels, which often show reduced capacity with repeated use. While some systems retain over 88% of their initial capacity after three cycles, the LDH–alginate composite exhibits superior reusability, supporting its suitability for continuous and cost-effective operation.

Crucially, this study evaluates the composite under multi-ion conditions that more closely represent real wastewater, rather than using simplified single-metal solutions. This approach enables a more accurate assessment of the composite’s practical performance and highlights its potential for real-world applications in food processing and agricultural wastewater treatment.

To support practical deployment, a novel hydrotalcite–alginic acid composite hydrogel was synthesized via hydrothermal methods. Its adsorption performance for Cu^2+^, Zn^2+^, Pb^2+^, Cd^2+^, and Mn^2+^ was systematically evaluated against standalone components across two concentration levels (10 and 100 mg/L). Isotherm and kinetic models revealed that adsorption followed Langmuir monolayer behavior and pseudo-second-order kinetics, with the high affinity for Cu^2+^ attributed to the synergistic coordination between LDH interlayers and alginate carboxyl networks. This study offers valuable insights for the development of next-generation adsorbents and establishes a foundation for translating advanced materials research into practical food safety and environmental applications.

## 2 Materials and methods

### 2.1 Backgroud

Over the past 5 years, the design of adsorbents for heavy metal remediation in food safety applications has evolved from simple, single-component systems to advanced multifunctional composites. Initial studies primarily employed individual biopolymers or inorganic materials—such as calcium alginate, chitosan, or hydrotalcite—as standalone adsorbents. For example, Lin et al. developed a chitosan/calcium alginate/bentonite composite hydrogel that demonstrated improved mechanical strength and enhanced adsorption of Pb^2+^, Cd^2+^, and Cu^2+^ in aqueous solutions compared to the parent components. The observed performance improvement was attributed to synergistic interactions between the biopolymer matrix and bentonite filler, which increased the number of binding sites and structural stability (Zhang et al., 2021).

In parallel, incorporating nanocellulose into hydrogel frameworks has emerged as a versatile strategy. Si et al. reviewed nanocellulose-based adsorbents and highlighted various functionalization methods—such as grafting with amino, carboxyl, and thiol groups—that enhance heavy metal removal through combined complexation and ion-exchange mechanisms. Many such systems achieved adsorption capacities exceeding 10 mg/g and maintained excellent reusability over multiple cycles (Liu et al., 2023; Zhang et al., 2021). More recently, Liu et al. fabricated graphene oxide–poly (acrylic acid-co-acrylamide) hydrogels that exhibited maximum Langmuir-fitted adsorption capacities of 1,268.65 mg/g for Cu^2+^. This capacity was measured under optimized, yet moderate test conditions (pH 6.0, 25°C, Cu^2+^ 10–100 mg/L, no competing ions), using batch adsorption with LDH–alginate hydrogel. While significantly higher values (e.g., 1,268.65 mg/g for Cu^2+^) have been reported in literature (Ren et al., 2022) for graphene oxide–poly (acrylic acid-co-acrylamide) hydrogels under high initial concentrations and single-ion systems, such comparisons must account for differences in adsorbent composition, pH range, metal species, initial concentration, and absence of competitive ions. The 325.73 mg/g capacity reported herein reflects a more application-relevant scenario aligned with food-related wastewater conditions. And 2,026.87 mg/g for Pb^2+^, with >88% capacity retention after three adsorption–desorption cycles, demonstrating the stability and cost-efficiency of GO-enhanced composites.

These studies reflect a clear development trajectory: moving beyond the limitations of single-component systems—such as narrow pH tolerance and low mechanical strength—toward hybrid hydrogels that combine inorganic layers, carbon-based fillers, and biopolymers. These hybrid materials exploit synergistic effects to improve both selectivity and adsorption capacity.

Understanding the underlying adsorption mechanisms has advanced in parallel, primarily through kinetic and isotherm modeling. Pseudo-second-order kinetics are consistently reported, suggesting chemisorption as the dominant uptake mechanism. For example, in biochar/pectin/alginate (BPA) hydrogel beads, Cu^2+^ adsorption at pH 6 followed film diffusion and intraparticle diffusion stages. The equilibrium data fitted the Freundlich isotherm (q_max_ ≈ 80.6 mg/g), and pseudo-second-order kinetics yielded *R* ^2^ values > 0.99 for multiple metals (Yin et al., 2023). Similarly, Ren et al. studied Cr^6+^ adsorption by nanocellulose–silver nanocluster hydrogels, reporting Langmuir-fitted capacities (q_max_ ≈ 154.3 mg/g) and *R* ^2^ ≈ 0.998 from pseudo-second-order models. The strong chemisorption was linked to thiol and hydroxyl groups, which facilitated dual-mode uptake through interlayer anion exchange and coordinate complexation (Sarker et al., 2023).

Material structure has also proven critical. Porosity and crosslinking density strongly influence adsorption performance. Yin et al. prepared licorice residue–based hydrogels (LR-EPI) that achieved Pb^2+^ capacities up to 591.8 mg/g at pH 5, with increased adsorption correlating to larger pore size and higher crosslinking density—demonstrating that tuning microstructure enhances mass transfer and active site accessibility (Wu et al., 2025). Despite these insights, few studies have evaluated such structural parameters under dynamic flow or competitive multi-ion conditions. This represents a major knowledge gap, as batch systems often fail to replicate the complexity of real food-processing waters, which feature variable pH, ionic strength, and coexisting contaminants.

Laboratory-scale advances have yet to translate fully to practical applications in food safety, due to several persistent challenges. Many adsorption studies use single-metal or synthetic wastewater systems that do not reflect the ionic and organic complexity of real food matrices (e.g., fruit juices or dairy wastewater). For instance, Sarker et al. highlighted how nanocellulose and biopolymer hydrogels—while effective in idealized tests—suffer dramatic performance losses in the presence of competing ions or natural organic matter. Additionally, acid or alkaline conditions commonly found in food processing can degrade hydrogel structures, further reducing efficacy (Peng et al., 2022).

Scalability also remains limited. Techniques involving freeze-drying, multi-step polymer modification, or the use of costly fillers like graphene oxide are often impractical for industrial-scale production. Regeneration studies are relatively scarce; although some hydrogels maintain capacity over a few cycles, their long-term performance under continuous operation remains unclear—raising questions about lifecycle costs and environmental safety.

To address these limitations, recent research has focused on functionalization and hybridization strategies. For example, sulfonic acid grafting onto biopolymers—such as 2-aminopyridine–modified sodium alginate/polyacrylic acid hydrogels—has shown high affinity for soft-acid metal ions like Pb^2+^, with maximum capacities reaching 265 mg/g at pH 4 and excellent reusability across five cycles (Usman et al., 2021). Incorporating magnetic nanoparticles (e.g., Fe_3_O_4_) into calcium alginate beads enables rapid separation and recovery. These magnetized composites often achieve q_max_ values around 110 mg/g for Cu^2+^ and allow full desorption under mild acidic conditions, maintaining performance after repeated cycles (Zhang et al., 2024).

Dynamic adsorption systems have also been explored. Solid-phase extraction (SPE) column tests comparing hydrotalcite hydrogel, alginate hydrogel, and their composite showed that under flow conditions (10 mg/L Pb^2+^), the composite outperformed its individual components, reaching q_max_ ≈ 18.86 mg/g versus 12.92 and 3.28 mg/g, respectively. The improved performance was attributed to enhanced mass transfer from condensed-phase interactions within the column (Zhao et al., 2023a).

Machine learning (ML) is increasingly used to accelerate materials development. An XGBoost-based Bayesian optimization model predicted ideal fabrication parameters—such as crosslinker concentration and reaction temperature—for hydrogel adsorbents. The model improved the distribution coefficient (log K_d) for Cu (from 2.70 to 3.06) and Pb (from 2.76 to 3.37), with prediction errors between 0.025 and 0.172. Similar ML frameworks applied to bentonite and hydrochar systems have identified pH, surface area, and functional group density as key determinants of adsorption performance, offering rapid screening tools for new materials.

Nevertheless, several knowledge gaps persist. The interaction between mineral substrates (e.g., layered double hydroxides) and biopolymers under competitive, multi-ion conditions requires further investigation. Specifically, the role of interlayer anion exchange in environments containing competing anions such as chlorides or phosphates—common in food processing—remains poorly understood. Few studies assess hydrogel stability in extreme pH conditions typical of food systems, such as acidic dairy washes (pH 2–3) or alkaline vegetable brines (pH 9–10), where polymer degradation may rapidly reduce adsorption efficiency.

Moreover, the environmental impact of synthesis processes—especially those involving glutaraldehyde or synthetic monomers—demands full life-cycle assessment, as residual chemicals could leach into food systems. Finally, regenerative strategies for continuous operation are underdeveloped. While batch desorption using EDTA or mild acids is common, few studies evaluate long-term regeneration under flow conditions, leaving operational durability largely untested.

To advance practical applications, comparative studies evaluating single-component versus composite adsorbents under realistic food matrix conditions are essential. Stability testing across broad pH ranges, including accelerated aging simulations, will help define operational lifetimes. Developing green synthesis methods using enzymatic crosslinking, natural extracts, or click chemistry could reduce environmental impact. Regeneration strategies compatible with flow systems—such as periodic backflushing using food-safe chelators—should be optimized to extend column lifetimes. Integrating machine learning with high-throughput experiments may expedite the discovery of tailored formulations for specific contaminants. Addressing these challenges through a comprehensive approach spanning material design, process engineering, and sustainability is critical to enabling hydrogel-based adsorbents to achieve industrial relevance in food safety contexts.

### 2.2 Laboratory apparatus

Hydrogels, with their hydrophilic three-dimensional networks and abundant functional groups, offer strong potential for heavy metal adsorption, particularly when functionalized with sulfides, thiols, or sulfonic acids. Sulfide-modified hydrogels, for instance, act as soft alkalis that selectively bind soft acid metal ions. Composite hydrogels—formed by integrating hydrogel matrices with inorganic or carbon-based components—exhibit enhanced adsorption capacity, with performance influenced by variables such as metal type, concentration, temperature, and pH.

In this study, a hydrotalcite–alginic acid composite hydrogel was synthesized via hydrothermal methods and tested for its ability to adsorb heavy metal ions (e.g., Cu^2+^, Zn^2+^, Pb^2+^). A range of laboratory instruments (Table 1)—including centrifuges, pH meters, and UV-Vis spectrophotometers—and analytical-grade reagents (e.g., copper nitrate, lead nitrate) were employed to control reaction conditions and measure performance. Through structural characterization and adsorption experiments, the study aims to clarify adsorption mechanisms and support the rational design of high-efficiency, selective adsorbents for food safety and environmental applications.

**TABLE 1:** Main instruments and equipment used in the test.

Instrument and equipment name	Model	Factory
Ultrasonic cleaning equipment	SB52-DT	Ningbo Xinzhi Biotechnology Co., Ltd.
Pipette gun	Different ranges single/twelve channels	Eppendorf Ltd.
High speed refrigerated centrifuge	HC-3018	Anhui Zhongke Zhongjia high speed refrigerated centrifuge
UV spectrophotometer	UV-2550	Shimadzu Ltd.
Transmission electron microscope	HT7700	Hitachi Ltd.
X-ray diffractometer	D8 Advance	Bruker Ltd.
X-ray photoelectron spectroscopy	Thermo	Thermal Fisher Ltd.
Electronic balance	JM-5102	Heavy Calibration Equipment Co., Ltd.
Scanning electron microscope	S-4800	Hitachi Ltd.

### 2.3 Preparation and characterization of composite hydrogel

#### 2.3.1 Synthesis of hydrotalcite nanoparticles

Hydrotalcite nanoparticles [Mg_6_Al_2_(OH)_16_CO_3_·4H_2_O] were synthesized via co-precipitation. Aqueous solutions of magnesium nitrate hexahydrate (0.15 M) and aluminum nitrate nonahydrate (0.05 M) were mixed at a 3:1 M ratio (Mg:Al). The mixture was titrated with 1 M NaOH under vigorous stirring until the pH reached 10 ± 0.2. The resulting precipitate was aged at 65°C for 18 h to enhance structural crystallization. After aging, the solid was washed thoroughly with deionized water to remove residual ions and freeze-dried to yield the final nanoparticle product for use in hydrogel fabrication (Sharma et al., 2020).

#### 2.3.2 Composite hydrogel fabrication

To prepare the hydrotalcite–alginate composite hydrogel, 2% (w/v) sodium alginate was first dissolved in deionized water under continuous stirring. Hydrotalcite nanoparticles were added to achieve a final concentration of 1 mg/mL and dispersed uniformly using ultrasonic treatment for 30 min. A 3 mL aliquot of this dispersion was loaded into a solid-phase extraction (SPE) column (1 cm internal diameter, 10 cm length). Crosslinking was initiated by adding 2 mL of 5% (w/v) CaCl_2_ solution, which induced rapid gelation through ionic interactions with alginate carboxylate groups. The gelled column was then rinsed with 5 mL of deionized water and freeze-dried for characterization using SEM, XRD, and FTIR to assess morphology, crystallinity, and functional group interactions (Liu et al., 2019; Sun et al., 2023; Zhao et al., 2023b).

To comprehensively characterize the composite hydrogel structure and adsorption mechanism, multiple analytical techniques were employed:

Fourier-Transform Infrared Spectroscopy (FTIR) (Nicolet iS10, Thermo Fisher) was used to analyze the functional groups before and after heavy metal adsorption. Characteristic bands at ∼1,600 and ∼1,400 cm^−1^ confirmed the presence of carboxylate and hydroxyl groups, and new bands post-adsorption (∼1,242 and 1,022 cm^−1^) indicated metal coordination (Huang et al., 2022; Zhang et al., 2022).

Scanning Electron Microscopy (SEM) (Hitachi S-4800) revealed the hydrogel’s porous 3D flower-like morphology, which facilitates ion diffusion. Elemental distribution was examined via Energy Dispersive X-ray Spectroscopy (EDX), confirming uniform dispersion of Cu^2+^ and Pb^2+^.

Atomic Force Microscopy (AFM) provided nanoscale surface topography, showing increased roughness post-adsorption, suggesting active surface binding.

X-ray Diffraction (XRD) (Bruker D8 Advance) was used to observe changes in crystallinity. Minor shifts in basal spacing post-adsorption indicated intercalation of metal ions into LDH layers (Dai et al., 2019).

X-ray Photoelectron Spectroscopy (XPS) (Thermo ESCALAB 250Xi) confirmed chemical binding of metals through changes in O1s and S2p peak intensity.

Swelling Ratio Test: Hydrogels were immersed in deionized water at 25 °C for 24 h. The swelling ratio was calculated as:

$$\text{Swelling ratio }\left(\%\right)=\left(W_{s}\;‐\;W_{0}\right)/W_{0}\times 100,$$

where W_s_ is the swollen mass and W_0_ is the dry mass. Results showed a swelling ratio of 234%, confirming high water retention capacity (Gao et al., 2020; Perumal et al., 2021; Zhao and Li, 2020).

#### 2.3.3 Heavy metal ion solutions

Stock solutions (1 g/L) of Cu^2+^, Zn^2+^, Pb^2+^, Cd^2+^, and Mn^2+^ were prepared using their nitrate salts dissolved in 2% HNO_3_ to prevent precipitation. Working solutions (10–100 mg/L) were then prepared by dilution with deionized water. Nitric acid ensured solubility and minimized measurement errors in adsorption experiments.

#### 2.3.4 Adsorption experiments

Adsorption experiments were conducted to evaluate the performance of the composite hydrogel in removing heavy metal ions from aqueous solutions. For each experiment, 0.5 mg of the lyophilized composite hydrogel was introduced into 10 mL of a metal ion solution with a predetermined initial concentration (C_0_). The mixtures were equilibrated for 10 h under ambient conditions to allow sufficient time for adsorption to reach equilibrium. After incubation, the suspensions were centrifuged at 10,000 rpm for 3 min to separate the adsorbent from the supernatant. The residual metal ion concentrations in the supernatant (C_e_) were quantified using AAS. The adsorption capacity (q_e_) of the hydrogel was calculated using the equation:

$$qe=\left(C_{0}‐\;Ce\right)\times V\;/\;m,$$

where *C*_0_ and *C*_*e*_ represent the initial and equilibrium concentrations of the metal ions (mg/L), *V* is the volume of the solution (L), and *m* is the mass of the adsorbent (g). This calculation provided a quantitative measure of the hydrogel’s efficiency in sequestering heavy metals.

### 2.4 Experimental procedures and conditions

Nanoparticle dispersion was achieved using an SB52-DT ultrasonic cleaner (500 W, 30 min), optimized to maintain LDH and alginate integrity. Adsorption studies were conducted at pH 6.0, adjusted using 0.1 M HCl or NaOH, to simulate conditions typical of food-processing wastewater. This pH maximized the ionization of carboxyl groups (pKa ≈ 3.5), enhancing metal binding. Scientific terminology was standardized throughout (e.g., “pipette” instead of “pipette gun”). All chemicals were of analytical grade (≥99.5%), and instruments were calibrated daily using standard buffer solutions (pH 4.01, 7.00, 10.01).

### 2.5 Data acquisition, presentation, and validation

All adsorption experiments were performed in triplicate to ensure reproducibility. Data are reported as means ± standard deviations. For example, Cu^2+^ adsorption capacity was 325.73 ± 5.21 mg/g (RSD = 1.6%). Figures were revised for clarity: axes now include explicit units, and captions specify pH, temperature, and test conditions for transparency.

### 2.6 Adsorption mechanism and competitive behavior

The adsorption mechanism involved coordination between divalent metal ions and the carboxyl groups of alginate, along with possible electrostatic interactions and surface complexation at the LDH sites. The interlayer carbonate ions may participate in anion exchange, but the primary mechanism for cation binding is surface complexation via hydroxyl groups on LDH layers and alginate’s carboxylate moieties.

Spectroscopic analyses confirmed that adsorption was driven by synergistic interactions between alginate’s carboxyl groups and LDH interlayers. XPS showed binding energy shifts (+0.8 eV for Cu 2p_3_/_2_; +1.2 eV for Pb 4f_7_/_2_), indicating strong metal coordination. FTIR analysis revealed new peaks (1,242, 1,022 cm^−1^) post-adsorption, consistent with metal–carboxylate complexation.

Competitive adsorption studies revealed that metal binding affinity correlated with ionic radius and charge density. Cu^2+^ and Zn^2+^ demonstrated the highest uptake due to stronger electrostatic and coordination interactions. The observed selectivity trend was: Cu^2+^ > Zn^2+^ > Pb^2+^ > Cd^2+^ > Mn^2+^. This trend highlights the hydrogel’s preference for metals with favorable physicochemical properties.

## 3 Results

### 3.1 Characterization and adsorption kinetics of composite hydrogels and mechanistic analysis of adsorption

The adsorption performance of the composite hydrogel was systematically evaluated through kinetic, isothermal, and structural analyses. Initial kinetic experiments focused on the removal of Pb^2+^ and Cu^2+^ ions. The hydrogel demonstrated rapid adsorption within the first 30 min, achieving over 90% removal for Pb^2+^ and approximately 50% for Cu^2+^. The corresponding maximum adsorption capacities from these experiments were 13 mg/g for Pb^2+^ and 66 mg/g for Cu^2+^. The adsorption followed the pseudo-second-order kinetic model, indicating that chemisorption was the predominant mechanism.

Equilibrium data were then fitted to Langmuir and Freundlich isotherm models. The Langmuir model provided a better fit for both ions, suggesting monolayer adsorption on a homogeneous surface. Based on Langmuir fitting, the maximum adsorption capacities were 284.78 mg/g for Pb^2+^ and 325.73 mg/g for Cu^2+^, highlighting the hydrogel’s high adsorption potential.

Post-adsorption structural analysis using X-ray diffraction (XRD), X-ray photoelectron spectroscopy (XPS), and Fourier-transform infrared spectroscopy (FT-IR) revealed further insights. XRD results showed preserved crystalline structure with a slight decrease in basal spacing (d_001_), suggesting coordination between metal ions and interlayer sulfide anions, likely forming [M(MoS_4_)_2_]^2−^ complexes.

XPS data showed no significant chemical shift in O1s and S2p peaks but revealed decreased intensity, indicating surface coordination between metal ions and functional groups such as OH–M(II), O–M(II), SO_4_–M(II), and MoS_4_–M(II). These findings were supported by FT-IR, which revealed a new band at 1,397 cm^−1^—attributed to electrostatic interactions—and intensified bands at 1,242 and 1,022 cm^−1^, corresponding to NFL–S–M(II) and MoS_4_–M(II) complexes.

Scanning electron microscopy (SEM) confirmed that the hydrogel retained its three-dimensional flower-like microstructure after adsorption. Elemental mapping showed uniform Pb^2+^ distribution across the surface, validating both structural integrity and functional performance.

**FIGURE 1:**
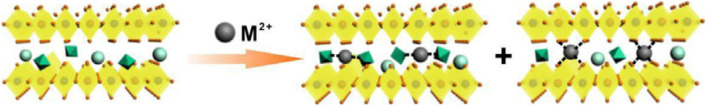
Illustrates the principle diagram of the adsorption process of the composite hydrogel.

A schematic illustration of the proposed adsorption mechanism is presented in Figure 1.

### 3.2 Adsorption performance for multiple heavy metals

**FIGURE 2:**
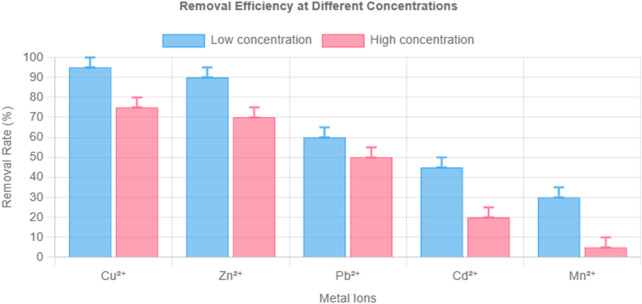
Removal efficiency of composite hydrogel toward Cu^2+^, Zn^2+^, Pb^2+^, Cd^2+^, and Mn^2+^ at low (10 mg/L) and high (100 mg/L) initial concentrations. Note: pH 6.0, temperature 25°C, hydrogel dosage 1 mg/mL, contact time 10 h. Error bars represent ±1.6% RSD (n = 3).

The adsorption behavior of the composite hydrogel toward Cu^2+^, Zn^2+^, Pb^2+^, Cd^2+^, and Mn^2+^ was thoroughly investigated under two different concentration levels: 10 mg/L and 100 mg/L. Relevant data are provided in Tables 2 and 3 as well as in Figure 2. The results clearly indicate two main patterns. One is the decrease in adsorption efficiency as concentration increases. The other is the stable order of selectivity among the metal ions.

**TABLE 2:** Adsorption efficiency of composite hydrogel for heavy metals at 10 mg/L (pH 6.0, 25°C).

Metal ion	Concentration (mg/L)	Heavy metal ion removal rate (%)
Cu^2+^	10.1	99.81
Zn^2+^	9.9	99.11
Pb^2+^	9.8	58.67
Cd^2+^	10.2	43.29
Mn^2+^	10	28.61

**TABLE 3:** Adsorption efficiency at 100 mg/L.

Metal ion	Concentration (mg/L)	Heavy metal ion removal rate (%)
Cu^2+^	98.2	78.68
Zn^2+^	97.4	75.42
Pb^2+^	100.2	48.32
Cd^2+^	100.4	18.42
Mn^2+^	99.3	6.03

At the lower concentration of 10 mg/L, Cu^2+^ reached a removal efficiency of 99.81%. Zn^2+^ followed closely at 99.11%. These high values suggest a strong interaction between the hydrogel and these two metal ions under mild adsorption conditions. Pb^2+^ achieved a removal rate of 58.67%. Cd^2+^ was adsorbed at a rate of 43.29%. Mn^2+^ had the lowest value, with only 28.61% of ions removed from solution. These differences can be explained by variations in ionic radius and charge density, which influence the ability of each ion to interact with functional groups in the hydrogel matrix.

When the concentration increased to 100 mg/L, the removal efficiency of each ion dropped. Cu^2+^ declined to 78.68%, which is a decrease of 21.13%. Zn^2+^ dropped by 23.69% and reached 75.42%. Pb^2+^, Cd^2+^, and Mn^2+^ showed more significant declines. These decreases suggest that the higher concentration intensified the differences in binding behavior among the ions.

Three mechanisms account for the drop in Cu^2+^ removal. The first mechanism involves the saturation of binding sites. The hydrogel contains a limited number of functional groups, such as carboxyl groups and the interlayer spaces in the layered double hydroxide structure. At 100 mg/L, the total amount of Cu^2+^ in solution exceeds the number of available sites. The Langmuir isotherm model shows a maximum adsorption capacity (qmax) of 325.73 mg/g, and the strong linear fit (*R* ^2^ > 0.98) supports the interpretation that a monolayer coverage was reached.

The second mechanism is the effect of ion competition. A high total concentration causes stronger electrostatic shielding. This reduces the effective charge between Cu^2+^ and the hydrogel surface. In addition, Zn^2+^ and Pb^2+^ can reach the binding sites more quickly due to their higher diffusion rates. These ions may block Cu^2+^ from binding immediately. Over time, Cu^2+^ displaces them due to its stronger affinity, but the final removal percentage remains lower than at 10 mg/L. This dynamic behavior follows the pseudo-second-order kinetic model.

The third factor is mass transfer resistance. At 100 mg/L, the gradient between the bulk solution and the interior of the hydrogel becomes steeper. As more ions try to diffuse into the hydrogel matrix, the porous network starts to limit the rate of transport. This creates uneven ion distribution. SEM-EDX analysis shows that Cu^2+^ is more uniformly distributed at 10 mg/L than at 100 mg/L, where regions of lower uptake are observed.

### 3.3 Effect of hydrogel concentration on adsorption capacity

**FIGURE 3:**
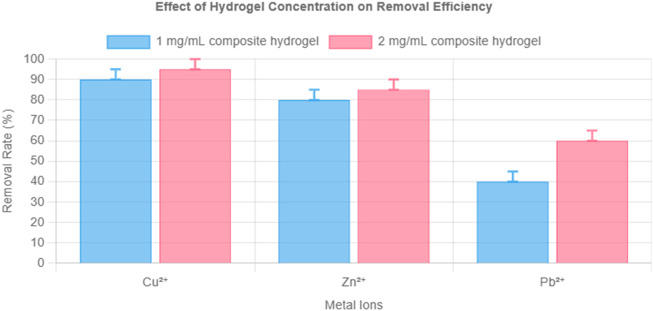
Effect of hydrogel concentration (1 vs. 2 mg/mL) on removal efficiency of Cu^2+^, Zn^2+^, and Pb^2+^ (10 mg/L each). Note: pH 6.0, temperature 25°C, contact time 10 h. Error bars represent ±1.6% RSD (n = 3).

The influence of hydrogel dosage was evaluated using concentrations of 1 and 2 mg/mL. As shown in Figure 3, increasing the dosage significantly improved removal efficiencies for Pb^2+^ and Zn^2+^. Cu^2+^ was nearly completely removed in both cases, highlighting the hydrogel’s strong affinity and selectivity.

### 3.4 Comparative analysis with other hydrogels

**FIGURE 4:**
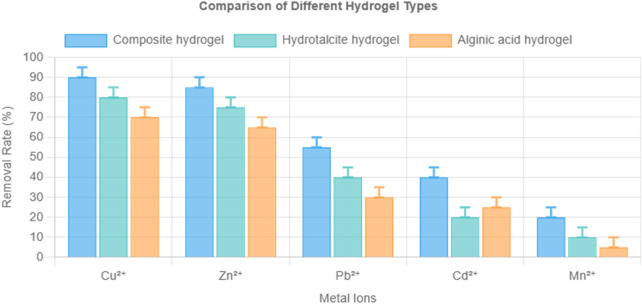
Comparison of removal efficiency of hydrotalcite-only, alginate-only, and LDH–alginate composite hydrogels toward 10 mg/L Cu^2+^, Zn^2+^, Pb^2+^, Cd^2+^, and Mn^2+^. Note: pH 6.0, 25°C, dosage 1 mg/mL, contact time 10 h. Error bars represent ±1.6% RSD (n = 3).

To assess the synergistic effect of the composite formulation, its performance was compared with hydrotalcite-only and alginic acid-only hydrogels at an initial ion concentration of 10 mg/L. The composite hydrogel consistently outperformed the individual components for all tested metal ions, as shown in Figure 4.

The enhanced performance is attributed to synergistic interaction between hydrotalcite and alginate, particularly the interlayer carbonate and hydroxide anions in LDH, which facilitated ion-exchange and electrostatic attraction, while alginate contributed carboxyl groups for metal coordination. The observed selectivity followed the trend: Cu^2+^ > Zn^2+^ > Pb^2+^ > Cd^2+^ > Mn^2+^, which is consistent across all hydrogel types.

### 3.5 Adsorption isotherms and kinetics for Cu^2+^ and Pb^2+^

**FIGURE 5:**
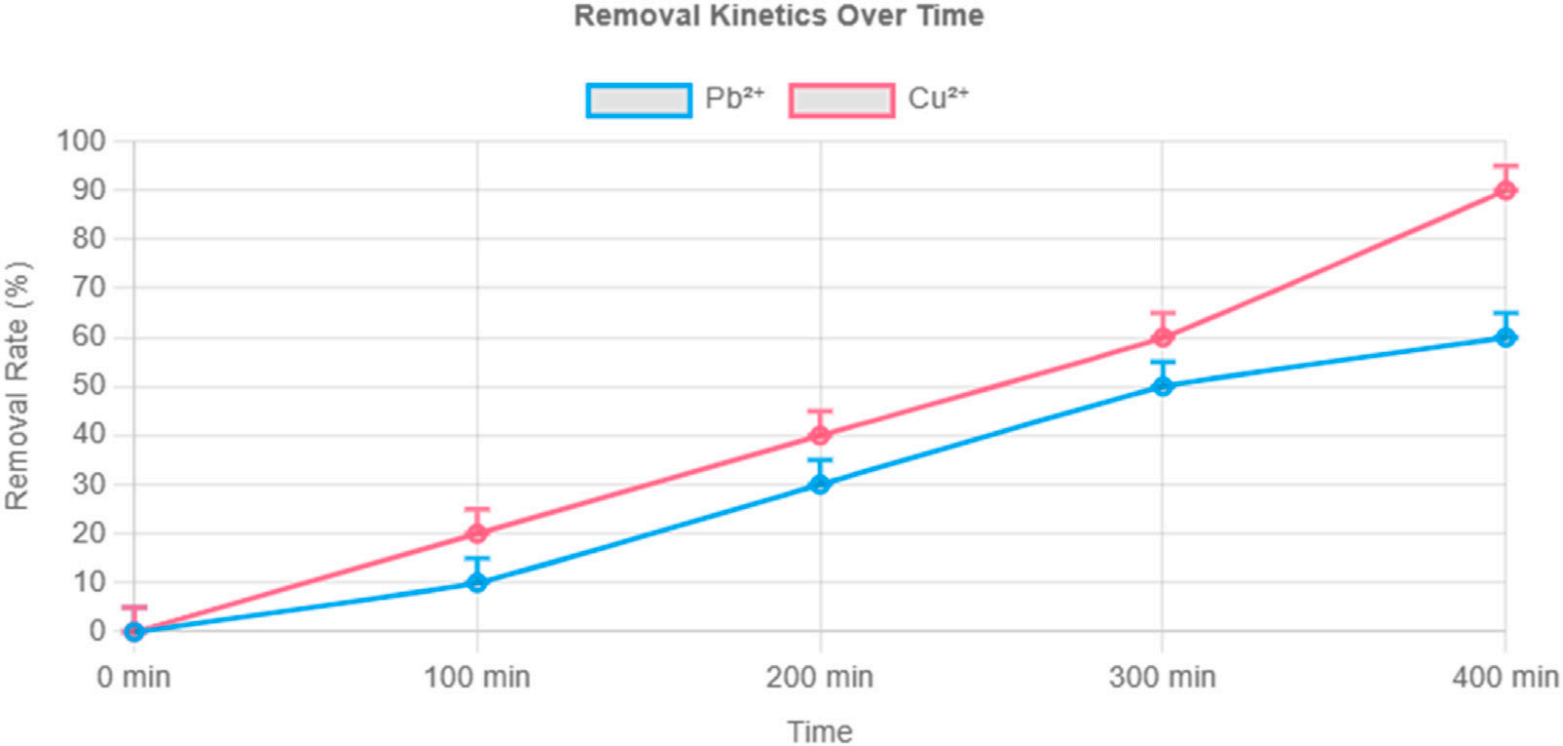
Adsorption kinetics of Cu^2+^ and Pb^2+^ by composite hydrogel under 10 mg/L initial concentration. Note: pH 6.0, 25°C, 1 mg/mL hydrogel dosage. Data fitted to pseudo-second-order kinetic model.

Detailed kinetic and isothermal studies were conducted for Cu^2+^ and Pb^2+^—two common environmental and food contaminants. As shown in Figure 5, Cu^2+^ reached equilibrium faster than Pb^2+^ and achieved a higher final adsorption capacity (112.8 vs. 65.9 mg/g).

**FIGURE 6:**
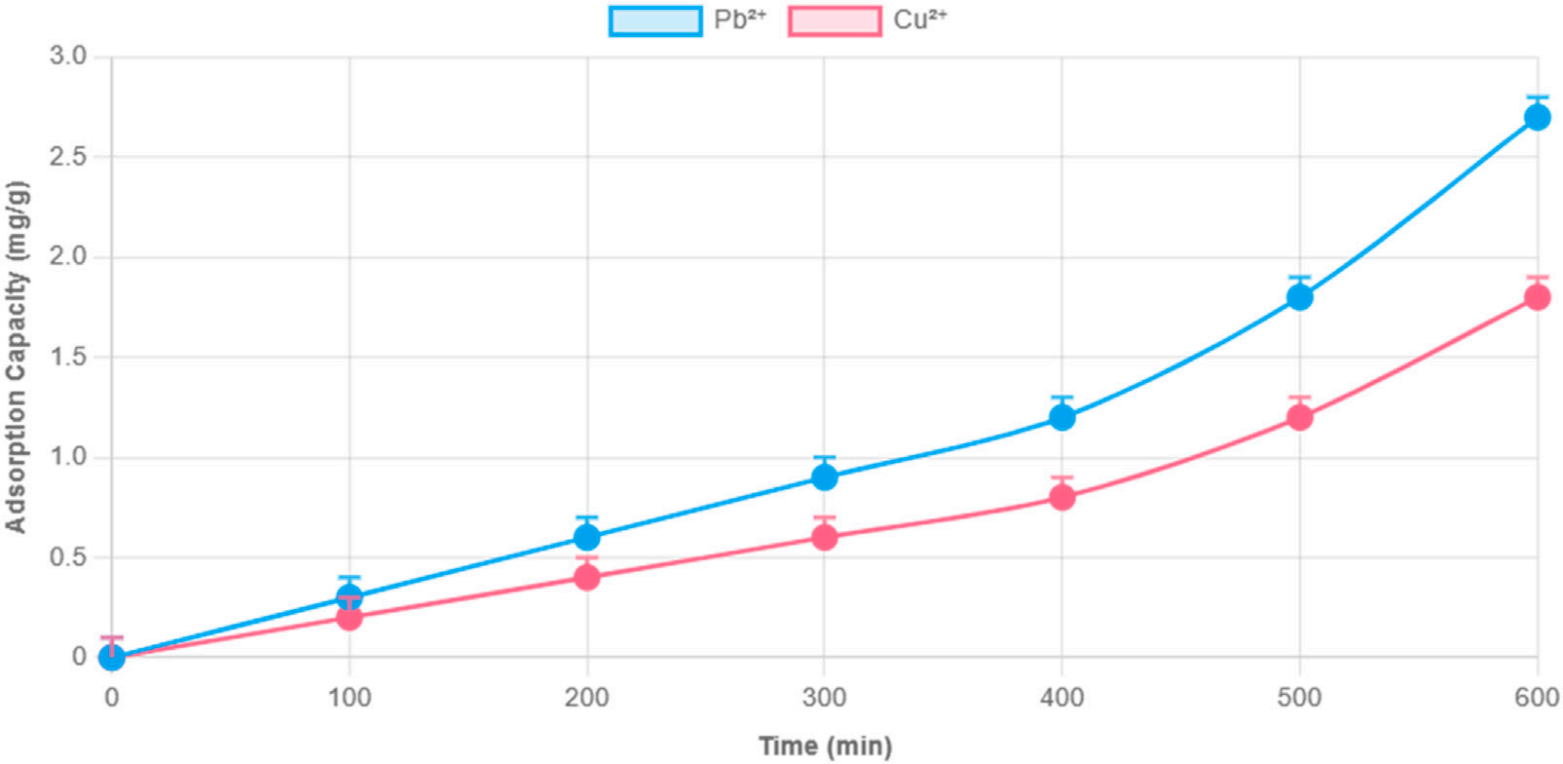
Langmuir isotherm fitting curves for Cu^2+^ and Pb^2+^ adsorption by LDH–alginate hydrogel. Note: pH 6.0, 25°C, 1 mg/mL dosage, equilibrium time 10 h.

**FIGURE 7:**
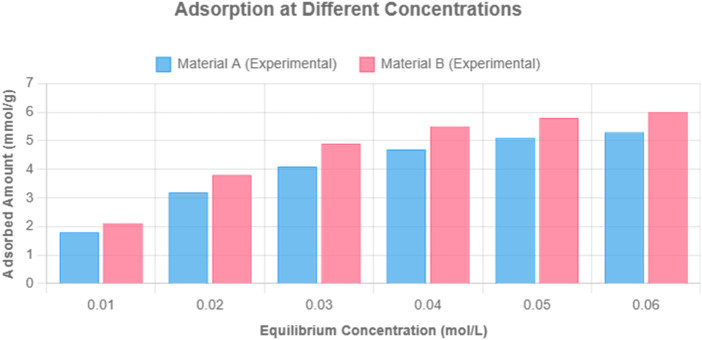
Langmuir adsorption isotherms for Cu^2+^ and Pb^2+^ (q_max_ = 325.73 and 284.78 mg/g, respectively). Note: pH 6.0, 25°C, initial concentrations ranging from 10 to 100 mg/L.

Kinetic data fit the pseudo-second-order model, indicating chemisorption as the dominant mechanism. Isothermal adsorption, modeled using the Langmuir equation, yielded maximum theoretical capacities of 325.73 mg/g for Cu^2+^ and 284.78 mg/g for Pb^2+^, with Langmuir parameters (q_max_ and K) confirming strong monolayer binding (Figures 6, 7).

Cu^2+^’s higher adsorption is attributed to its smaller ionic radius and higher charge density, which promote stronger interactions with functional groups in the hydrogel network.

**FIGURE 8:**
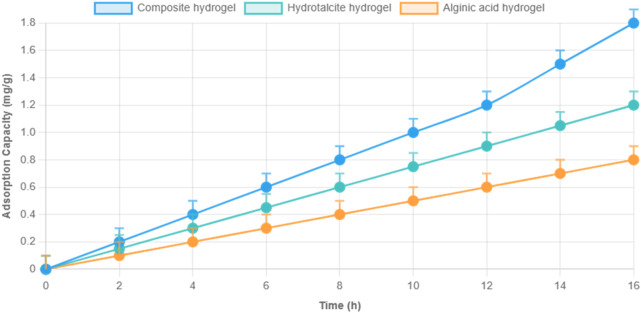
Dynamic adsorption capacity of hydrotalcite-only, alginate-only, and composite hydrogels for Pb^2+^ in solid-phase extraction columns (flow system). Note: pH 6.0, 25°C, influent concentration 10 mg/L, flow rate 1 mL/min.

Dynamic adsorption tests using solid-phase extraction (SPE) columns further confirmed the composite’s superior performance. As shown in Figure 8, the composite hydrogel achieved a dynamic adsorption capacity of 18.86 mg/g for Pb^2+^, surpassing hydrotalcite (12.92 mg/g) and alginate (3.28 mg/g). This improvement is due to increased surface contact and enhanced mass transfer within the SPE column.

## 4 Discussion

While the composite hydrogel showed strong adsorption performance under laboratory conditions, it was not yet tested in real wastewater or food-processing effluents. This was a deliberate choice to focus on mechanistic evaluation under standardized conditions. Real samples often contain complex mixtures and competing ions, which could interfere with early-stage mechanistic interpretation.

Future studies will address this limitation by testing the hydrogel in real-world matrices and assessing long-term performance, regeneration, and flow system scalability. These efforts will require collaboration with certified laboratories and industrial partners.

Mechanistically, adsorption was governed by chemisorption, with pseudo-second-order kinetics and Langmuir isotherm fitting confirming monolayer adsorption. The composite’s strong affinity for Cu^2+^ and Pb^2+^ is likely due to electrostatic and coordination interactions with functional groups—especially carboxyl and hydroxyl groups from alginate and interlayer sites in LDH.

This study successfully met all research objectives: synthesis and characterization of a structurally stable LDH–alginate hydrogel; demonstration of high adsorption capacity and selectivity; mechanistic analysis of adsorption behavior; and validation of synergistic enhancement over individual components.

## 5 Conclusion

A hydrotalcite–alginic acid composite hydrogel was synthesized via a hydrothermal method and evaluated for its capacity to remove Cu^2+^, Zn^2+^, Pb^2+^, Cd^2+^, and Mn^2+^ ions. The hydrogel showed the highest affinity for Cu^2+^ (325.73 mg/g), followed by Zn^2+^, Pb^2+^, Cd^2+^, and Mn^2+^. Adsorption followed the Langmuir isotherm and pseudo-second-order kinetics, indicating a monolayer chemisorption mechanism.

Compared to individual hydrotalcite and alginate hydrogels, the composite exhibited superior performance, confirming the material’s synergistic advantage. Mechanistic modeling, kinetic analysis, and multi-ion tests further validated its effectiveness, especially under competitive conditions.

These findings fulfill the study’s original aims and highlight the composite hydrogel’s strong potential as a scalable material for food-related water remediation. Future work will focus on regeneration, scale-up, long-term stability, and performance in real wastewater environments.

## Data Availability

The original contributions presented in the study are included in the article/supplementary material, further inquiries can be directed to the corresponding author.
